# Attitudes of Health Care Professionals Toward Older Adults’ Abilities to Use Digital Technology: Questionnaire Study

**DOI:** 10.2196/26232

**Published:** 2021-04-21

**Authors:** Ittay Mannheim, Eveline J M Wouters, Leonieke C van Boekel, Yvonne van Zaalen

**Affiliations:** 1 School for Allied Health Professions Fontys University of Applied Science Eindhoven Netherlands; 2 Tranzo Tilburg School of Social and Behavioral Sciences Tilburg University Tilburg Netherlands

**Keywords:** ageism, attitudes, stereotype activation, digital technology

## Abstract

**Background:**

Digital technologies (DTs) for older adults focus mainly on health care and are considered to have the potential to improve the well-being of older adults. However, adoption rates of these DTs are considered low. Although previous research has investigated possible reasons for adoption and acceptance of DT, age-based stereotypes (eg, those held by health care professionals) toward the abilities of older adults to use DTs have yet to be considered as possible barriers to adoption.

**Objective:**

The aim of this study was to investigate the influencing role of ageism in the context of health care professionals attitudes toward older adults’ abilities to use health care DT. A further goal was to examine if social comparison and stereotype activation affect and moderate this association.

**Methods:**

A new measurement to assess health care professionals’ attitudes toward older adults using technology (ATOAUT-10) was developed and used in 2 studies. Study 1 involved the development of the ATOAUT-10 scale using a principal component analysis and further examined health care professionals’ attitudes toward the use of health care DTs and correlations with ageism. Study 2 further explored the correlation between ageism and ATOAUT in an experimental design with health care professionals.

**Results:**

In study 1, physiotherapists (N=97) rated older adults as young as 50 years as less able to use health care DT compared to younger adults (*P*<.001). A multiple regression analysis revealed that higher levels of ageism, beyond other predictors, were predictive of more negative ATOAUT, (β=.36; *t*=3.73; *P*<.001). In study 2, the salience of age was manipulated. Health care professionals (N=93) were randomly assigned to rate the abilities of a young or old person to use health care DT. Old age salience moderated the correlation between ageism and ATOAUT (*R*^2^=0.19; *F*_6,85_=3.35; *P*=.005), such that higher levels of ageism correlated with more negative ATOAUT in the old age salient condition, but not the young condition. Stereotype activation accounted for health care professionals’ attitudes more than did the experience of working with older patients or the professionals’ age.

**Conclusions:**

Negative and ageist attitudes of health care professionals can potentially affect how older adults are viewed in relation to DT and consequently might influence actual use and adoption of technology-based treatment. Future studies should broaden the validation of the ATOAUT-10 scale on more diverse samples and focus on the discriminatory aspect of ageism and self-ageism of older adults. This study calls for a focus on ageism as a determinant of adoption of DT.

## Introduction

Digital technology (DT), hereby defined as technological devices, services or platforms that use, collect, and often process data and are connected to the internet, other devices, or apps [1], are thought by many to have the potential to improve quality of life and promote independent and active aging of older adults [2-4]. However, older adults are often discoursed as a homogenous group of “nonusers”[5], associated with illness, frailty, cognitive decline, and dependency [6]. This might be one of the reasons why DTs developed for the use of older adults primarily focus on health care [2], which positions health care professionals in the forefront of using DT with older adults. Substantive research has attempted to explain the factors for adoption of DT by older adults in general [7-9] and specifically for health care [10]. Yet, the specific influence of age-based stereotypes and ageism on the use and adoption of DT in care and health care, and the effect of social comparison and stereotype activation on health care professionals’ attitudes toward older adults in relation to DT, have not been investigated.

Ageism comprises stereotypes, prejudice, and discrimination toward a person based on their age [11]. This definition reflects a cognitive component (eg, the belief that older adults are less able to use DTs), an emotional component (eg, the feeling that instructing older adults how to use DT is annoying), and a behavioral component (eg, not offering older adults treatments based on DT). The pervasiveness and social acceptability [12,13] of ageism is to some extent explained by its indirect and often implicit nature. Social behavior is often implicitly shaped by environmental cues and activation of stereotypical traits. Ageism is thus internalized throughout the life course and can operate implicitly [14] often without awareness as to how it influences our judgments [15]. Activation of age stereotypes and ageism, therefore, do not necessarily encompass explicit intention to do harm, as it is often expressed subtly in forms of benevolence [16], and older adults being disrespected, ignored, or patronized [17]. Therefore, it is important to measure both implicit and explicit attitudes [18]. Ageism can indeed be harmful and affect the opportunities of older adults for active aging, equal participation, and access to services such as health care [19] or DTs [20]. Furthermore, implicit attitudes and stereotypes are often embodied and self-directed [21] and may eventually lead to decreased physical and mental health [22].

Expressions of ageism manifest in different contexts and life domains, such as health care, leisure and employment, and different domains have different age thresholds as to when a person is considered “old” [23]. Ageism can also be context specific [24], meaning that negative attitudes toward older adults, (eg, use of DT) can vary in the context of family, work, or health care. A central stereotype about older adults that is very much apparent in relation to DT is that they are less competent [12], and simply presenting a question about “old” or “young” can lead to stronger associations with negative age traits [25].

Contrary to stereotypes that portray older adults as “laggards” [26], nonusers [5], and technophobic [27], accumulating evidence suggests that older adults find DT to be fascinating and empowering [28], and hold more positive than negative attitudes toward DT [29]. According to a recent report by the American Association of Retired Persons (AARP), use of various forms of DTs (eg, smartphones, tablets, smart home technologies) by adults aged 50 years and above has consistently increased since 2014, and for many devices, adoption rates are nearly similar to those of younger adults [30]. More importantly, reasons for which older adults use or do not use DT are complex and include social context, emotions, experience, support, and individual preferences [8,31], and perhaps also relate to social influence and attitudes of others [7], such as family members or health care professionals. Noticeably, there is often a mismatch between what is designed for the use of older adults (mainly in the context of health care) and what they actually want and need [32], which may be DT that is both enjoyable and empowering [33]. Older adults express high willingness to use certain care and health care DTs (eg, monitoring sensors), however, only if they perceive that their health status might severely decline [34]. Subsequently, this mismatch might lead to low adoption rates and abandonment of health care–related DT [10].

There is considerable evidence for ageism among health care providers, both self-reported and patient reported [35]. Negative age stereotypes, usually operating in indirect or implicit manners, were found to influence diagnosis, prognosis, and treatments provided. Ambady et al [36] found that implicit measures such as distancing and nonverbal communication of physiotherapists during treatment (eg, looking away from the person) were associated with short- and long-term physical decline of older patients. Patients with similar symptoms or complaints are often diagnosed differently or misdiagnosed because of their age. In a study by Linden and Kurtz [37], the same case description (differing only by age) led to a diagnosis of depression for younger patients and a diagnosis of dementia for older patients. Meanwhile, in a study by Gewirtz-Meydan and Ayalon [38], manipulating the age salient in a case description led to a different prognosis and treatment trajectory of a sexual function complaint. Unfortunately, use of chronological age in triage strategies (eg, in the Covid-19 pandemic) also raises ethical discussions about the influence of ageism on medical decisions [39]. As many aspects of health care are digitalizing, it remains unclear if these ageist manifestations might affect the use of DT with older adults.

There is contradicting evidence regarding characteristics of health care professionals that are associated with ageist outcomes [35]. Some studies indicate that negative attitudes are often associated with younger age of health care professionals [40], whereas positive attitudes are associated with older age, female professionals, and positive experience of working with older patients [41]. Other findings suggest that knowledge about aging and choosing to work with older adults might determine professionals’ attitudes and reduce stereotypes and prejudice [42]. The latter might fit in with the idea of social contact theory [43].

In their daily work, health care professionals need to categorize patients in order to reach practical medical decisions. This type of categorization might be considered functional [44]. However, with a view of stereotypes as a process of internalizing and learning [14], possible biases of health care professionals might be seen as consequence of increased exposure to older adults in situations of illness and dependency. This “clinician bias” might shape the general image health care professionals have of older patients, leading to “diagnostic overshadowing” [45] and pushing them to differentiate themselves from older patients, for example, because of existential fear of their own death [46]. It is therefore plausible that implicitly activated age stereotypes, created in situations of social comparison or categorization [47], determine health care professionals’ attitudes toward older adults, more than characteristics of age, gender, or experience. This might lead to the disregarding of individuating information, perception of older adults as a homogeneous outgroup, and discriminatory behaviors.

The aim of this study was to investigate whether ageism plays a role in the perception of health care professionals toward older adults’ abilities to use health care DT, or, in other words, whether people who reach a certain age are considered “too old” to use health care DT. Therefore, we first were interested in determining whether higher levels of ageism in health care professionals would be associated with negative attitudes regarding older adults’ abilities to use DT. Second, we looked into whether social comparison and stereotype activation would affect this association. Generally, we hypothesized that health care professionals’ attitudes of older adults’ abilities to use DT would be negative and that higher levels of ageism would be associated with more negative attitudes. Furthermore, we hypothesized that social comparison and stereotype activation would moderate this association, leading to attitudes that are more negative. In study 1 we developed and tested new measurement tools and subsequently assessed the association between ageism and attitudes toward older adults’ abilities to use DT. In study 2 we further tested how manipulating stereotype activation might moderate this association. Both studies received ethical approval from the Fontys University of Applied Science ethics research committee (approval file no. Mannheim22022019).

## Methods

### Study 1

The goal of study 1 was to initially assess the explicit and implicit attitudes of health care professionals toward older adults’ abilities to use DT and the association between these attitudes and ageism. As specific measures are currently unavailable, an additional goal was to develop and test the use of new direct and indirect measurements of DT-related attitudes and ageism. We hypothesized that health care professionals would express negative attitudes toward the abilities of older adults (compared to younger adults) to use DT. We further hypothesized that higher levels of ageism would be correlated with more negative attitudes.

### Participants

We recruited physiotherapists working in the Netherlands and fourth year physiotherapy students who had already gained professional experience during their internships using available mailing lists between April 2019 and May 2019. Out of the 155 who were contacted, 97 participants voluntarily completed the questionnaire. A statistical power analysis was performed for sample size estimation, using G*Power 3.1.9.4 (Heinrich Heine University Düsseldorf). We used the assumption of a minimal effect size of 0.2 (as no prior knowledge of this scale is available), with an α of .05 and a power of 0.9. The projected sample size needed for a multiple regression with 4 predictors was 82, 75% (73/97) of participants were female, M_age_ = 32.39 (SD 11.24), 23% (22/97) were fourth year students, 34% (33/97) had 1-5 years of work experience, 28% (27/97) had 6-20 years of experience, and the remaining 15% (15/97) exceeded 20 years of experience. Finally, 47% (46/97) indicated that most of their patients were 65 years or older.

### Measures

#### Ageism

The Fraboni Scale of Ageism (FSA) was used as a direct measure of ageism [48]. The FSA assesses all 3 dimensions of ageism: stereotypes, prejudice, and discrimination [11]. As no available translation in Dutch was available, the questionnaire was forward translated from English to Dutch by 2 assessors (YVZ and another assessor), and back translated to English by 2 different assessors (EJMW and another assessor). Differences in interpretation and culturally sensitive aspects were then discussed with all 4 assessors and the corresponding author (IM). The scale consists of 29 items ranked on a 4-point ordinal scale (1, strongly disagree; 2, disagree; 3, agree; 4, strongly agree). Reversed items were recoded and a sum score of the scale was calculated (scale range 29-116). Higher scores represent higher levels of ageism. Missing values in 4 items (7 missing values in total) were replaced by the mean of the item as previously suggested in the use of the FSA by Helmes and Pachana [49]. The Cronbach α coefficient of the scale was .85, similar to the reliability levels found by Fraboni et al [48].

#### Attitudes Toward Older Adults Using Technology

As a direct measure, we developed the attitudes toward older adults using technology (ATOAUT) scale. Items were developed in accordance with known literature about stereotypes on older adults and technology, such as ease of use and perceived benefit [7,29], fear, anxiety, and self-efficacy [50,51]; our experience from interviewing technology designers and focus groups with older adults [52]; and feedback from experts. We eventually reached a group consensus regarding 15 items that potentially assess stereotypes (eg, “Using digital technology is harder for most older adults”) and prejudice (eg, “One needs a lot of patience to explain to an older adult how to use digital technologies”) toward older adults and DT (for the full list of items see Table 1). Participants rated their agreement with statements about older adults and DT on a Likert-type scale from 1 (totally disagree) to 6 (totally agree). Five reversed items were recoded, and a sum score of the scale was calculated (scale range 15-90). Higher scores represent attitudes that are more negative.

As an indirect measure, we modified a vignette technique previously used to assess health care–related ageism [37,38]. Participants were presented with 3 descriptions of health care–related DTs, namely a health care app, smartwatches, and rehabilitation videogames (see Multimedia Appendix 1). Participants were then asked to rate (yes or no) if they believed different age groups (18-30 years, 31-50 years, 51-64 years, 65-79 years, and 80+ years) could use this DT. Positive answers were coded as 1 and negative answers as 0. Answers for each age category in all 3 vignettes were summed, creating a measure between 0-3 for each age group. The Cronbach α coefficient of all items was .82.

Additionally, we used a direct question to assess the belief that age might be a barrier to using DT. Participants rated (yes or no) if they believed that gender, age, culture, or financial situation can limit a person’s ability to use technology.

### Procedure

Participants received an invitation through email to participate in a study about how older adults use technology in health care and everyday life. Participants were directed to an online questionnaire on Qualtrics, where they gave consent to participate. Afterward, they answered questions about demographic information and to which age group most of their patients belonged. Following this, they answered the DT-related ageism measures (vignettes and ATOAUT scale) and the FSA.

Additionally, we inserted an unrelated validity item (“For this question only, mark the number 2”) in the middle of the FSA and ATOAUT scales to check actual reading of the question.

### Analysis

SPSS Statistics 26 (IBM Corp) was used to perform the analysis. In order to examine the ATOAUT scale, a principal component analysis was performed, and modifications were made to the scale. In order to examine our hypothesis on the attitudes of physiotherapists on the indirect measure of the vignettes, a repeated measures analysis of variance (ANOVA) was performed, with the sum of vignettes as the dependent variable, the age group assessed as the within-subject independent variable, primary age group of patients the physiotherapists work with as the between-subject independent variable, and the age of the physiotherapists as a covariate. Finally, to examine the correlation between ATOAUT and ageism (FSA scale) and other variables, we used a correlation matrix and a multiple regression.

### Study 2

The goal of study 2 was to test how age salience and stereotype activation might moderate the correlation between ageism and ATOAUT. Specifically, we wanted to address the limitations of study 1: that by merely asking all participants to rank the abilities of every age group to use DT, we actually primed social categorization [47] and age-based stereotypes. Therefore, we sought to control the age group salient in the vignettes, so that participants would need to rate the ability of a young or contrastively an old person to use the DTs described in the vignettes. Additionally, we sought to broaden our findings to a more diverse group of health care professionals. We hypothesized that older adults would be assessed as less able than younger adults in using health care DTs. Furthermore, we hypothesized that the “old” salience condition would prime age stereotypes and the need to categorize and differentiate oneself from the older group, leading to attitudes that are more negative. In contrast, rating the “young” condition would allow participants to affirm their self-concept without categorizing and comparing themselves to the older group. Therefore, we hypothesized that the age salient manipulation would act as a moderator in the correlation between ageism and ATOAUT.

### Participants

We recruited 93 health care professionals and fourth year health care students in the Netherlands between December 2019 and February 2020. A statistical power analysis was performed for sample size estimation, using G*Power 3.1.9.4 (Heinrich Heine University Düsseldorf). We used the assumption of a minimal effect size of 0.2, with an α of .05 and a power of 0.9. The projected sample size needed for a multiple regression with 6 predictors was 94. Participants were recruited among the students and staff at a university of applied science, and the questionnaire was further distributed within their networks of health care professionals. Of the respondents, 67% (62/93) of the participants were female, M_age =_ 37.01 (SD 11.89), 38% (35/93) were physiotherapists, 25% (23/93) were speech therapists, 17% (16/93) were medical doctors, and 20% (19/93) belonged to other health professions. Moreover, 18% (17/93) were students, 33% (31/93) had 1-5 years of experience, 29% (27/93) had 6-20 years, and the remaining 20% (18/93) had more than 20 years of experience. For patient age, 41% (38/92) of the professionals indicated that most of their patients are 65 or older.

### Measures

#### Ageism

Due to the limitations of the FSA in study 1, we used the Expectations Regarding Aging (ERA-12) scale [53]. Consisting of 12 items, the ERA-12 is a shorter and more updated scale compared to other available ageism scales and is considered to have the most adequate psychometric properties [11]. The items reflect stereotypes about aging in general and toward one’s own aging. As a Dutch translation to the ERA-12 was not available, we used the forward–backward translation method described in study 1. Items were ranked on a 4-point ordinal scale (1, definitely false; 2, somewhat false; 3, somewhat true; 4, definitely true). A summed score was calculated (scale range 12-48), with higher scores representing more negative expectations regarding aging. The Cronbach α coefficient of the scale was .78, which was slightly lower than that reported in Sarkisian et al [53].

#### Attitudes Toward Older Adults Using Technology

As a direct measure, we used the ATOAUT-10 scale developed in study 1 (for the factor analysis and description of how we reduced the scale from 15 to 10 items see the Results section). The Cronbach α coefficient was .73, which slightly lower than that in study 1.

For the indirect measure we used the vignettes developed in study 1 with certain modifications. We used 2 of the 3 vignettes from study 1 (health care app and smartwatch), and replaced the third (videogames in rehabilitation) as it was specific to the context of physiotherapists. Instead, we added a new vignette of using a voice-activated personal assistance, such as Siri, google personal assistant, or Alexa (see Multimedia Appendix 1). Participants rated the probability that a person (25 or 75 years old) would be able to use the described DT on a Likert-type scale between 1 (not at all) to 6 (very much so). The scores of the 3 vignettes were summed to create the total measure (scale range 3-18). The Cronbach α coefficient was .86.

### Procedure

Participants received an invitation through email to participate in a study about health care professionals’ perspectives about using DT in everyday life and in health care. Participants were directed to an online questionnaire on Qualtrics, where they gave consent to participate and answered demographic questions. Participants were then randomly assigned to rate one of the contrastive age groups (young or old) in the 3 vignettes, and then completed the ATOAUT-10 and ERA-12 scales, and the question used in study 1 about the belief of age being a limiting factor. Finally, participants indicated the percentage of their patients who are above the age of 65 years. One validity check item was inserted in the middle of the ATOAUT-10 scale.

### Analysis

**Figure 1:**
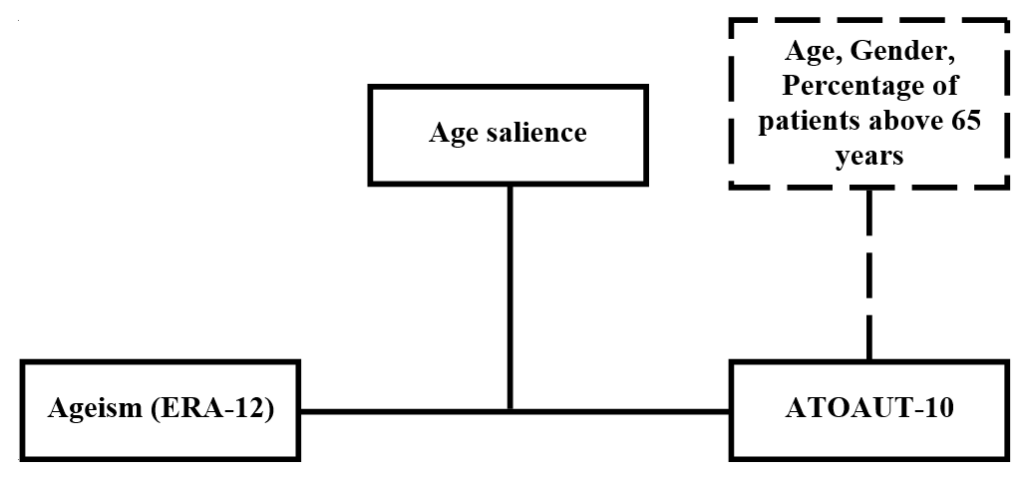
Assumed moderation model of age salience (young and old) on the correlation between ageism (measured by ERA-12) and attitudes towards older adults using technology (ATOAUT-10). Age, gender, and percentage of patients above 65 years were added to the model as covariates. ATOAUT: attitudes toward older adults using technology; ERA-12: Expectations Regarding Aging scale.

SPSS 26 (IBM Corp.**)** was used to perform the analysis. In order to examine our hypothesis that older adults would be assessed as less able than younger adults to use health care DTs on the indirect measure, we used a correlation matrix and multiple regression. In order to examine our hypothesis that age salience would moderate the correlation between ATOAUT and ageism (ERA-12), we employed a regression procedure using the PROCESS bootstrapping macro [54] in SPSS (model 1: 5000 iterations). Figure 1 presents the assumed moderation model.

## Results

### Study 1

We found that 27 participants did not answer the validity items correctly. Consequently, concern regarding their attention in answering was raised. Analysis including and excluding these participants, revealed that the results would not be different. We therefore included all 97 participants in this study.

### ATOAUT Scale

The initial Cronbach α coefficient of the 15-item scale was .77. We then used a principal component analysis in order to examine the latent components of the scale. Bartlett test confirmed no violation of sphericity (approximate χ^2^ = 379.1; *P*<.001). Five factors with eigenvalues larger than 1 were identified and accounted for 62.07% of the variance (see Table 1). Eight items loaded mainly on the first factor (all loadings above 0.43), representing stereotypes and prejudice toward older adults’ abilities to use DT (eg, “Using digital technology is harder for most older adults”), with the exception of item 2, were also strongly loaded on the fourth factor. Two additional items (5 and 12), loaded on the second factor, represented attitudes toward older adults’ access to DT and online digital services. Examining the remaining 5 items that did not load on the first and second factor revealed that the phrases used might have been ambiguous and interpreted variably. Consequently, answering them might not necessarily reflect stereotypes or prejudice toward older adults, but rather general attitudes toward the role of DT in improving well-being (item 8), matters of privacy (item 9), accessibility of the design (items 4 and 11), and how playful people of different ages are (item 3).

Item 10, which loaded on the first factor, seemed to have a weaker loading compared to the other items. This might have been related to confusion in the use of negation in this item. We therefore concluded to change the phrasing for future use to “Most older adults *can* give useful feedback about new digital technologies.” Finally we formed the new ATOAUT-10 scale, comprising items 1, 2, 5, 6, 7, 10, 12, 13, 14, and 15. The Cronbach α coefficient of the new 10-item scale was higher (.82), compared to the 15-item scale coefficient (.77), and explained 91.2% of the variance of the 15-item scale.

**Table 1:** Initial eigenvalues and explained variance and loadings after Varimax rotation of ATOAUT items as sorted by loading size (N=97).^a^

Items and factors		Rotated component matrix				
		1	2	3	4	5
Initial eigenvalues		4.28	1.55	1.28	1.19	1.01
Variance explained (%)		28.51	10.36	8.52	7.95	6.74
**ATOAUT^b^ item (loadings)**						
	15. One needs a lot of patience to explain to an older adult how to use digital technologies	.81	.01	–.12	–.02	–.20
	7. Using digital technology is harder for most older adults	.76	.19	.04	.22	.08
	1. It’s hard to explain to older adults how to use digital technology	.71	.06	.01	.21	.08
	6. Most older people do not see the benefits of using digital technology	.63	.38	.13	–.07	–.12
	14. Most older adults are not interested in learning about using new digital technology	.58	.51	.10	.02	.02
	13. Most older adults fear using digital technology because they believe they will break or ruin something	.47	.15	–.35	.11	.19
	10. Most older adults cannot give useful feedback about new digital technologies	.43	.19	.21	–.12	.07
	5. Most older people have less access to digital technology	.27	.80	–.03	–.02	.10
	12. Online services can be used by adults of any age (for example online banking or government services)^c^	.08	.60	–.18	.53	–.05
	4. When designing new digital technologies for older adults, older adults should take part in the design process^c^	.19	–.20	.83	–.03	.07
	8. Using digital technology can improve older adults well–being and health^c^	.00	.34	.62	.30	–.05
	9. Using digital technology can cause more harm to older adults’ personal safety and privacy compared to younger adults	.01	.02	.07	.76	–.04
	2. Most older adults can use digital technology just as well as younger adults^c^	.53	–.01	.07	.55	.07
	11. Digital technology for older adults should be designed in a way that is accessible and easy to use^c^	–.30	.02	.34	–.19	.74
	3. Video game devices are mainly for younger adults	.34	.05	–.34	.12	.69

^a^The final ATOAUT-10 scale comprised items 1, 2, 5, 6, 7, 10, 12, 13, 14, and 15.

^b^ATOAUT: attitudes toward older adults using technology.

^c^Reversed item.

### Indirect Measure of Attitudes: Vignettes

**Figure 2:**
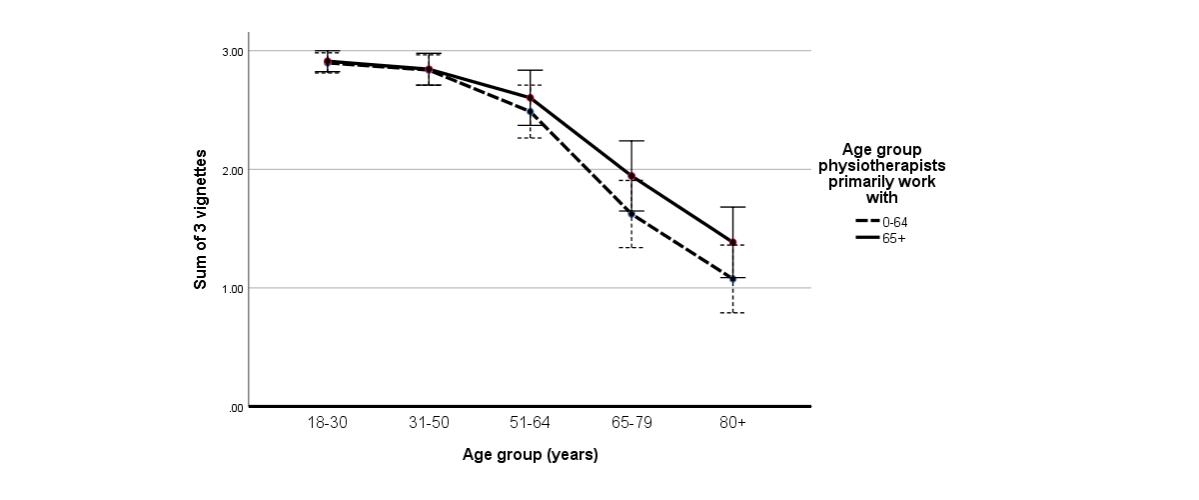
Assessed ability to use health care digital technologies by age group assessed and the primary age the physiotherapists work with (N=94). Error bars: 95% CI.

Table 2 presents the means, SDs, and correlations of the sum of the 3 vignettes for each age category, ATOAUT-10, FSA, and other variables. Physiotherapists assessed older age groups as less able to use the health care DTs described in the vignettes (Figure 2). We further examined these differences using a repeated measures ANOVA, with the sum of vignettes as the dependent variable, age group assessed as the within-subject independent variable, primary age group of patients the physiotherapists work with as the between-subject independent variable, and age of the physiotherapists as a covariate. By interpreting the Greenhouse-Geisser test (due to violation of sphericity), we found a significant main effect of the age group assessed (*F*_2.33, 211.74_=34.66; *P*<.001; ηp^2^=0.28). Post hoc comparisons using the Bonferroni adjustment revealed that each age group above 31-50 years was assessed as significantly less able to use health care DT than the younger age group before it (all *P* values <.001). The interaction of age group assessed and age of the physiotherapists reached significance (*F*_2.33,211.74_=5.79; *P*=.002; ηp^2^=0.06), such that younger physiotherapists assessed the ability of older adults to use the DTs as lower than that of older physiotherapists. The interaction between age group assessed and the primary age group of patients the physiotherapists work with was not significant.

**Table 2:** Means, SDs, and correlations of the sum of the 3 vignettes for each age category, ATOAUT-10, FSA, and other variables (N= 95-97)^a^.

Variable	Mean (SD)	1, *r*	2, *r*	3, *r*	4, *r*	5, *r*	6, *r*	7, *r*	8, *r*	9, *r*	10, *r*
1. ATOAUT-10^b^	35.45 (8.45)	—^c^									
2. FSA^d^	50.62 (7.98)	.38**	—								
3. Gender^e^	0.75 (0.43)	.03	–.02	—							
4. Age	32.39 (11.25)	–.18	–.17	.12	—						
5. Primary age group physiotherapists work with^f^	0.47 (0.50)	.10	.05	.02	–.04	—					
6. Belief that age is a barrier to use of DT^g,h^	0.68 (0.47)	.48**	.13	.07	–*.*25*	.08	—				
7. Sum of 3 vignettes for 18-30 years age group	2.88 (0.36)	.17	.09	.13	.07	.09	.13	—			
8. Sum of 3 vignettes for 31-50 years age group	2.83 (0.45)	–.11	.08	.11	.02	.02	.04	.52**	—		
9. Sum of 3 vignettes for the 51-64 years age group	2.54 (0.78)	–.18	.06	.14	.12	.08	–.06	.28**	.64**	—	
10. Sum of 3 vignettes for the 65-79 years age group	1.77 (1.05)	–.35**	–.05	.16	.32**	.15	–.17	.06	.33**	.64**	—
11. Sum of 3 vignettes for the 80+ years age group	1.22 (1.02)	–.34**	–.22*	.22*	.21*	.14	–.10	.04	.27**	.51**	.76**

^a^2-tailed significant levels presented.

^b^ATOAUT: attitudes toward older adults using technology. Higher score represents more negative attitudes.

^c^Not applicable.

^d^FSA: Fraboni Scale of Ageism. Higher score represents higher levels of ageism.

^e^This variable was dummy coded (0 = male, 1 = female).

^f^This variable was dummy coded (0 = 0-64, 1 = 65+).

^g^DT: digital technology.

^h^This variable was dummy coded (0 = no, 1 = yes).

*P<.05.

**P<.01.

### Correlation Between Ageism and ATOAUT

As seen in Table 2, a more negative ATOAUT score was significantly correlated with higher levels of ageism (FSA), a belief that age can be a barrier to using DT, and lower perceptions of older adults’ abilities to use health care DTs (sum of vignettes for the 65-79 years and 80+ years age groups). Additionally, higher scores of ageism as measured by the FSA were correlated with lower perceptions of older adults’ abilities to use health care DTs (80+ years age group). To further examine the correlation between ATOAUT and ageism, we performed a multiple regression with ATOAUT-10 as the dependent variable, and FSA, age, gender, and the primary age group physiotherapists work with as independent variables. A significant model was found (adjusted *R*^2^=0.14; *F*_4,92_=4.75; *P*=.002), indicating that beyond all predictors, FSA was the only significant predictor (β=.36; *t*=3.73; *P*<.001).

### Study 2

One participant did not answer the validity item correctly. Analysis that included this participant revealed that the results would not be altered, and the participant was hence included in the analysis. Table 3 presents the means and SDs of the main variables by condition and correlations. No significant differences were found between participants in the different age salient conditions (young or old) regarding background variables of age, gender, health care profession, and percentage of patients over the age of 65 years that the professional works with. We then analyzed the indirect measure of assessing the ability in using actual health care DTs, using a multiple regression with the sum of the vignettes as the dependent variable and age salience condition and background variables (age, gender, and percentage of patients over the age of 65 years) as independent variables. As observed in Table 3, younger adults were perceived as more likely to use the described DTs compared to older adults. This was qualified by a significant regression model (adjusted *R*^2^=0.59; *F*_4,87_= 34.15; *P*<.001), with the age salience condition being the only significant predictor (β=–.78; *t* =–11.61; *P*<.001).

**Table 3:** Means and SDs by age salience (young and old) and correlations (N=93)^a^.

Variable	Total (N=93), mean (SD)	Young (n=49), mean (SD)	Old (n=44), mean (SD)	1, *r*	2, *r*	3, *r*	4, *r*	5, *r*	6, *r*	7, *r*
1.Age salience^b^	—^c^	—	—	—		—				
2. ATOAUT-10^d^	36.16 (6.50)	35.73 (6.96)	36.64 (6.00)	.07	—					
3. ERA-12^e^	28.27 (5.10)	27.61 (5.18)	29.00 (4.96)	.14	.06	—				
4. Sum of vignettes	12.72 (3.66)	15.41 (2.28)	9.73 (2.35)	–.78**	–.15	–.14	—			
5. Age	37.01 (11.89)	36.31 (12.15)	37.80 (11.69)	.06	–.31**	.11	–.05	—		
6. Gender^f^	0.67 (.47)	0.61 (.49)	0.73 (.45)	.12	–.03	–.06	–.07	–.06	—	
7. Percentage of patients over the age of 65 years that the professional works with^g^	39.02 (27.66)	38.16 (29.63)	40.00 (25.54)	.03	.20	.26*	–.01	–.11	.01	—
8. Belief that age is a barrier to use of DT^h^	0.73 (.45)	0.69 (.47)	0.77 (.42)	.09	.42**	.03	–.09	–.36**	–.07	.08

^a^2-tailed significant levels presented.

^b^This variable was dummy coded (0 = young, 1 = old).

^c^Not applicable.

^d^ATOAUT: attitudes toward older adults using technology. A higher score represents a more negative attitude.

^e^ERA: Expectations Regarding Aging. A higher score represents a higher level of ageism.

^f^This variable was dummy coded (0 = male, 1 = female).

^g^n=92.

^h^This variable was dummy coded (0 = no, 1 = yes).

*P<.05.

**P<.01.

**Figure 3:**
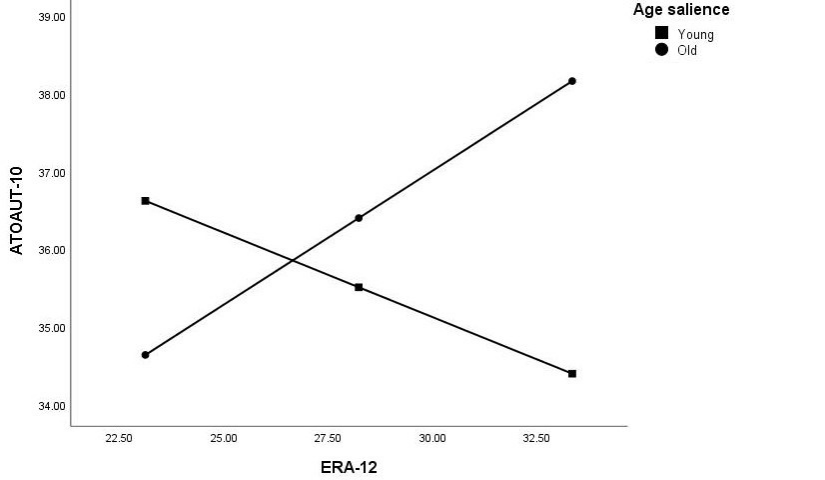
Moderation of the correlation between ATOAUT and ageism (ERA-12) by age salience (N=92). ATOAUT: attitudes toward older adults using technology; ERA-12: Expectations Regarding Aging.

Examining the direct measure of the ATOAUT-10 scale revealed that negative ATOAUT scores correlated with the younger age of the health care professionals and belief that age is a barrier to using DT. Yet, the simple correlation with ageism (ERA-12) was insignificant. We then tested our hypothesis that age salience would moderate the correlation between ATOAUT and ageism. Table 4 and Figure 3 present the regression coefficients and interaction of age salience X ERA-12. A significant moderation model was found (*R*^2^=0.19, *F*_6,85_=3.35; *P*=.005) and the age salience X ERA-12 interaction qualified as a significant moderator, adding significant explanatory variance to the model (*R*^2^ change=0.05; *F*_1,85_=4.90; *P*=.03). For the conditional effect of the old age salience condition, negative ERA-12 (higher ageism) was (marginally) associated with a more negative ATOAUT score (β=.34; *t*=0.82 *P*=.07; CI –0.03 to 0.72), whereas for the young age salience condition, it was not (β =–.22; *t*=–1.23; **P*=.22*; CI –0.57 to 0.13). Moreover, the age of the professional was identified as a significant predictor of ATOAUT score, with the younger age of the professional being associated with a negative ATOAUT score.

**Table 4:** Regression coefficients of the moderation model between ERA-12 and attitudes toward older adults using technology (ATOAUT-10) by age salience, with controlling for age, gender, and professionals’ percentage of patients above 65 (N=92).

Variable	Coefficient	SE	*t*	*P* value	LLCI^a^	ULCI^b^
Constant	46.82	5.12	9.14	<.001	36.63	57.00
ERA-12^c^	–0.22	0.18	–1.23	.22	–0.57	0.13
Age salience^d^	–14.97	7.31	–2.05	.04	–29.51	–0.43
Age salience X ERA-12	0.56	0.25	2.21	.03	0.06	1.07
Age	–0.17	0.06	–3.14	.002	–0.28	–0.06
Percentage of patients above 65 years	0.04	0.02	1.74	.09	–0.01	0.09
Gender^e^	–0.74	1.36	–0.54	.59	–3.43	1.96

^a^LLCI: lower level confidence interval.

^b^ULCI: upper level confidence interval.

^c^ERA-12: Expectations Regarding Aging. A higher score represents a higher level of ageism.

^d^This variable was dummy coded (0 = young, 1 = old).

^e^This variable was dummy coded (0 = male, 1 = female).

## Discussion

### Principal Findings

The aim of these studies was to explore attitudes of health care professionals toward older adults’ abilities to use DT, as a specific domain of ageism. In study 1 we developed the ATOAUT-10 scale, a direct measurement of stereotypes and prejudice toward use of DT by older adults, and an indirect measurement (vignettes), which were thereafter used in study 2. In the process of developing the ATOAUT-10, we identified ten items that represent stereotypes and prejudice toward older adults in the context of using DT. As hypothesized, significant correlations were found between negative ATOAUT and higher levels of ageism, measured by 2 different ageism scales (FSA in study 1 and ERA-12 in study 2). More so, negative ATOAUT correlated with lower perception of older adults’ abilities to use actual health care DTs, measured by the vignettes; belief that age is a barrier to using DT; and younger age of the health care professionals (in study 2). Thus, enhancing its construct validity. The correlation with ageism measured by the FSA in study 1 was significant and accounted for additional predictors in a multiple regression. Yet, this correlation was small to medium, suggesting that ageism alone does not fully explain physiotherapists’ attitudes toward older adults’ abilities to use DT.

By using an indirect measure of the vignettes, we were able to assess health care professionals’ perceptions on how they believe older adults use health care DT. Our main finding in study 1, that individuals from older age categories are perceived as less able to use DTs is not surprising [10]. Yet still, the difference found between age groups was quite dramatic, with a significant difference between each age group above 31-50 years with the previous age group. Hence, a DT-specific age threshold [23] for negative assumptions about the ability to use health care DTs, might be as early as 50 years, which includes people in their working age who are considered to use DT on a daily basis, and not only people above 80 years. This finding alone might confirm an assessment that is based on stereotypes and not facts, as the majority of older adults in some countries use smartphones and the internet [30]. In study 1, rating all age groups by the participants might have activated age stereotypes by means of categorization and social comparison [47], but in study 2, the age group salient in the vignettes was manipulated (young or old). Once more, a significant difference was found in how the technological abilities of older adults were perceived by health care professionals. More importantly, as hypothesized, this subtle manipulation of age salience moderated the correlation between ageism and ATOAUT, such that higher levels of ageism were associated with a more negative ATOAUT score but only when old age was made salient beforehand. Although the moderator of age salience was significant, it should be noted that the effect size of the moderator’s addition to the explained variance was small.

These results reveal perspectives that are quite ageist, considering the accumulating evidence on the increasing prevalence of using DT by older adults. Furthermore, they demonstrate how DT-specific ageism can operate implicitly by merely inducing social comparison or making the concept of age salient. Consequently, this raises the concern that older adults might be discriminated in how they receive (or do not receive) technology-based treatments, as found in other studies with nontechnological treatments [37,38]. This is worrying, considering the discussed benefits of DT in facilitating health care and reducing costs [2]. Notably, we did not explicitly ask health care professionals about their intentions to offer DT-based treatments; therefore, the behavioral and discriminatory aspect of ageism was not addressed in this study. However, it can be assumed that attitudes and beliefs might influence intentions to use and actual use, as is often emphasized by technology acceptance models [7,9,10]. Interestingly, the correlation between ageism and ATOAUT was not significant for participants in the young age salient condition. This could be explained by the matching of the age category (young vs old) and the specific attributes of the context (eg, competency of older adults in using DT) that occurs only in the old age salient condition and may activate specific age-based stereotypes [55]. Although people might be unaware of the underlying processes that influence their attitudes, there is still a matter of controllability of induced behavior and actual use of stereotypes [15]. Explicit and implicit stereotypical evaluations are both, to some extent, prone to belief-based learning processes [18]. Therefore, control over behavioral expressions (namely discrimination) is also a matter of social norms and social acceptability. As ageism is still relatively socially acceptable, expressions of discrimination, especially in domains where older adults are highly stereotyped, such as DT, might be prevalent.

Although previous studies reported gender and more experience of treating older patients [41] as possible predictors of attitudes of health care professionals, these effects on ATOAUT or ageism were not found in this study. Younger age of the professional was found to predict negative attitudes only to a limited extent. Unfortunately, these findings do not shed new light into the inconclusive findings in the literature [35] and do not strongly support the idea that increased social contact with older adults [43] can reduce negative attitudes in relation to health care DTs. This might be due to the higher exposure of health care professionals to older adults who are ill or suffer from chronic conditions [45]. Subsequently, these findings suggest that stereotype activation might be a stronger predictor of negative ATOAUT score. However, the effect sizes found were relatively low. Perhaps a stronger manipulation of stereotype activation, such as priming negative age stereotypes, could lead to stronger effects. Alternatively, other characteristics should be taken into account, such as professionals’ desire to work with older adults, the valence of their professional and social contact with older adults, or previous experience of using DT in health care.

Knowledge and training are important aspects when trying to control or combat automatic stereotype activation. Gawronski et al [56] found that training people to acknowledge information that contradicts stereotypes may reduce automatic stereotype activation. Training might offer positive outcomes in reducing ageism in health care and enhance positive contact [57]. Nevertheless, formal training of health care professionals regarding ageing (more so stereotype activation) is still lacking in curricula, and the esteem of working with older patients is still low [58]. We therefore suggest that training should include modules to raise awareness on biases and how easily our behavior is affected by them.

This study focused mainly on the perspective of health care professionals. It is however important to consider that indirect expressions of ageism, including patronizing speech [17], “Elderspeak” [59] or other nonverbal communications [36], might in turn lead to stereotype activation within older patients. These stereotypes are often embodied [21] and directed toward oneself, further affecting participation [19] and actual use of DTs. Hence, attitudes of older adults, as well the reciprocal nature of the interaction between them and others, should be a focus of future research on DT-related ageism.

### Limitations

The findings of this study provide a preliminary basis for future research and validation of the ATOAUT-10 scale. However, there were 3 main limitations regarding our measurements. First, our sample size in study 1, while sufficient for the regression analysis, can be considered to be on the lower boundary of minimal sample sizes for factor analysis [60]. Furthermore, the sample in study 1 consisted only of physiotherapists. The sample in study 2, therefore, was broadened to a wider range of health care professionals. However, in order to expand the scale’s validity, future studies focusing solely on the validation of the scale with an appropriate sample size and including a diversity of health care professionals and other stakeholders, is needed. Second, it can be claimed that our sample was biased in gender and experience. Although early career and experienced professionals were represented in both studies, a better balanced and planned sampling might have enhanced the validation of the scale. Third, in study 1, a large number of participants missed the validity check item inserted in the FSA, which raised concerns they did not answer the FSA seriously enough. This could be due to the length of the FSA, and some outdated items which might be less suited to Dutch society. A recent review by Ayalon et al [11] found that the psychometric properties of the FSA are considered low compared to other ageism scales. This was the reason we used a different ageism scale in study 2 (ERA-12). Therefore, continued use of the ERA-12 in future studies aimed at replicating these findings is warranted. Finally, as mentioned, our study did not focus on the actual intentions of professionals to use health care DT, or, in other words, the behavioral aspect of discrimination and adoption of DT. This would also be a recommendation for future research with this scale, which could also be used to broaden theoretical models of technology adoption [7,9,10].

### Conclusions

The highly negative attitudes of health care professionals toward older adults’ abilities to use DTs raise the question of how DTs are used in treatment. Activation of age-based stereotypes seems to play a pivotal role in these attitudes, possibly suggesting that nonadoption of DT is not entirely attributable to chronological age or individual characteristics. Using health care DTs indeed presents an opportunity to improve treatment of chronic diseases and well-being. However, when categorizing the lower access of older adults to use DTs and discussing the digital divide as a purportedly well-established fact [28], we are often preserving a negative view that might hamper the successful implementation of DTs in health care. Instead, it is essential to acknowledge how the field of DT is constantly evolving and includes individuals from all ages with different wants and needs. It is suggested that in order to increase adoption of DT, the focus ought to shift toward how we can change stereotypes and their activation on the individual and societal level.

## Appendix

Multimedia Appendix 1 Vignettes used in study 1 and 2.

## Abbreviations

AARP: American Association of Retired Persons
ANOVA: analysis of variance
ATOAUT: attitudes toward older adults using technology
DT: digital technology
ERA-12: Expectations Regarding Aging scale
FSA: Fraboni Scale of Ageism
