# Supplementary material for: Attitudes of Health Care Professionals Toward Older Adults’ Abilities to Use Digital Technology: Questionnaire Study
Source: J Med Internet Res. 2021 Apr 21;23(4):e26232. doi: 10.2196/26232 (PMC8100887; doi:10.2196/26232)
Supplement: Multimedia Appendix 1 [file jmir_v23i4e26232_app1.docx]

### Appendix A: Vignettes used in study 1 & 2

Instructions: In the next section you will be presented with descriptions of new digital technologies that can potentially assist us in our daily life and improve our wellbeing.

**Study 1:** For each description you will be asked to indicate with which age categories of patients you think you can use these digital technologies (yes/no)? (Mark all relevant age categories)

18-30, 31-50, 51-64, 65-79, 80+

**Study 2 ‘young’ condition:** For each description you will be asked to indicate the probability that a 25 year old person will be able to use this technology, between 1 (not at all) to 6 (very much so).

**Study 2 ‘old’ condition:** For each description you will be asked to indicate the probability that a 75 year old person will be able to use this technology, between 1 (not at all) to 6 (very much so).

**Vignette 1:**

New healthcare Apps allow patients to receive personalized exercises from health care professionals (such as nurses, doctors, speech therapists or physiotherapists), and send back information about physical and health indicators. Patients need to install this app on their mobile phone in order to use and access this app.

Study 1 – If you would have access to this new application, with which age categories of patients do you think you can use this? (Mark all relevant age categories yes/no) 18-30, 31-50, 51-64, 65-79, 80+

Study 2 - How probable is a 25/75 year old person to be able to use this technology (1-6)?

**Vignette 2:**

Smartwatches are becoming more and more popular. They can also help in tracking daily activity and monitor health related indicators such as number of steps a day, heart rate, blood pressure and sleep patterns. This can promote a healthy life style and help to identify and decrease the risk of diseases such as cardiovascular diseases.

Study 1 – If you would have unlimited number of smartwatches to borrow to your patients, with which age categories of patients do you think you can use this? (Mark all relevant age categories yes/no) 18-30, 31-50, 51-64, 65-79, 80+

Study 2 - How probable is a 25/75 year old person to be able to use this technology (1-6)?

**Vignette 3 (study 1 only):**

Your working place just bought a video game device (Nintendo Wii, PlayStation, X box), that allows to use interactive games for therapy purposes. These games propose exercises that helps to increase the stability and balance of the participant. If you would have access to these new games, with which age categories of patients do you think you can use this? (Mark all relevant age categories yes/no) 18-30, 31-50, 51-64, 65-79, 80+

**Vignette 4 (study 2 only):**

Virtual personal assistance devices such as “Siri”, "google personal assistant" or “Alexa”, allow people to search for information, order products and services and even activate and control smart home objects (lights, temperature, cameras etc.). These devices can operate using voice commands, so that people do not have to actively hold the device or understand completely how it works.

How probable is a 25/75 year old person to be able to use this technology (1-6)?
